# Relationship between elevated soluble CD74 and severity of experimental and clinical ALI/ARDS

**DOI:** 10.1038/srep30067

**Published:** 2016-07-22

**Authors:** Guosheng Wu, Yu Sun, Kang’an Wang, Zhengli Chen, Xingtong Wang, Fei Chang, Ting Li, Ping Feng, Zhaofan Xia

**Affiliations:** 1 Department of Burn Surgery, Changhai Hospital, Second Military Medical University, 168 Changhai Road, Shanghai 200433, P. R. China

## Abstract

CD74 is expressed on the cell surface of pulmonary macrophages and contributes to macrophage migration inhibitory factor (MIF)-induced inflammatory response in acute lung injury (ALI). A circulating form of CD74 (soluble CD74, sCD74) was recently discovered in autoimmune liver disease. Using two murine ALI models and cells culture, we examined the presence of sCD74 in circulation and alveolar space and preliminarily assessed the biological function of sCD74. The concentrations of sCD74 were increased in serum and bronchoalveolar lavage fluids (BALF) of murine ALI models. The elevated levels of sCD74 in BALF positively correlated with lung permeability and inflammation. In addition, sCD74 is secreted by macrophages in response to MIF stimulation and itself can stimulate the production of inflammatory cytokines. Our clinical study confirmed some findings of basic research. Moreover, we also found Day 3 serum sCD74 levels were associated with worse clinical outcomes. In conclusion, higher serum sCD74 levels may reflect more severe lung injury and may be used to help physicians determine prognosis of acute respiratory distress syndrome (ARDS).

Acute lung injury (ALI) or acute respiratory distress syndrome (ARDS) is a life threatening condition due to direct or indirect injury. Numerous studies have shown ALI/ARDS is characterized by an inflammatory response in lungs associated with various inflammatory cytokines^1^. Increasing evidence supports that the cytokine known as macrophage migration inhibitory factor (MIF) plays an important role in leading to alveolar inflammation in ALI/ARDS and represents a potential biomarker in ALI/ARDS because it could augment pro-inflammatory cytokine secretion (TNF-α) and anti-MIF treatment effectively suppressed the level of neutrophil chemokines in the lungs^2^,^3^,^4^,^5^. In 2003, the invariant chain (Ii) expressing on cell surface was reported as a high-affinity membrane receptor for MIF^6^.

Ii is a nonpolymorphic type II integral membrane protein and acts as a molecular chaperone of major histocompatibility complex (MHC) class II^7^. Mature mouse Ii consists of a 29 amino acid (aa) cytoplasmic domain, a 29 aa transmembrane segment, and a 224 aa extracellular domain (ECD) that contains one thyroglobulin type I domain^8^. Alternate splicing generates a short isoform that lacks the thyroglobulin domain. It is known that about 2–5% of cellular Ii is expressed on cell surface given the name as CD74^9^,^10^. And cell surface CD74 was found in diverse cell types including monocytes, B cells, activated T cells, and fibroblasts^11^,^12^. Additionally, in the lung, surface CD74 expression was reported in macrophages, type II pneumocytes, and endothelial cells under hypoxia stimulation^13^,^14^,^15^. Recently, a study shows that CD74, expressing on the cell surface of pulmonary macrophages, contributes to the MIF-induced neutrophils accumulation into the alveolar space^14^. However, the overall role of CD74 in pulmonary inflammation remains largely unclear. Since a soluble form of CD74 (sCD74) was identified in serum of patients with autoimmune liver disease^16^, we hypothesized that sCD74 existed in circulation or alveolar space under ALI/ARDS pathological conditions and elevation of serum sCD74 would be associated with severity of ALI/ARDS.

The initial objectives of this study were to investigate whether sCD74 can be detected in serum and bronchoalveolar lavage fluids (BALF) and whether the levels of sCD74 in serum could reflect the severity of experimentally induced ALI. For this purpose, we used a mouse model of lipopolysaccharide (LPS)-instillation induced ALI and a mouse model of cecal ligation and puncture (CLP) induced ALI. The second objectives were to measure sCD74 in serum from patients with ARDS and examine the relationship of serum sCD74 levels to clinical outcomes in these patients. Serum sCD74 levels in patients with ARDS were also compared with those in normal healthy volunteers. Some results of this study have been previously reported in the form of abstract^17^.

## Results

### CD74 expression in murine lung tissues was increased following direct and indirect injury

**Figure 1:**
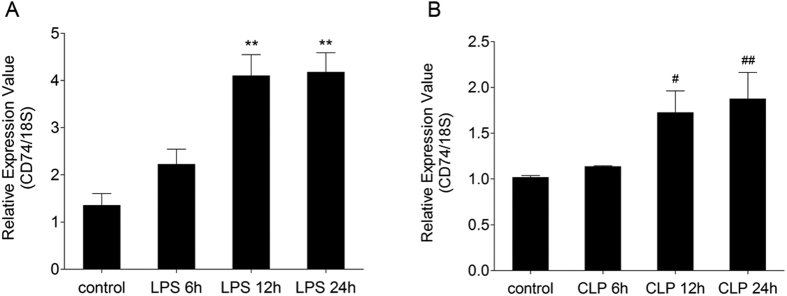
Quantitative Real-time PCR analysis of CD74 mRNA in lungs. Quantitative real-time PCR revealed a significant up-regulation of CD74 mRNA at 6, 12, 24 hrs in lipopolysaccharide induced lung injury (**A**) and cecal ligation and puncture induced lung injury (**B**). Quantitative real-time PCR data are representative of experiments performed in triplicate. n = 5 in each group. Data are presented as mean ± SEM, ^#^p < 0.05 and **, ^##^p < 0.01 compared to control with Dunnett-*t* test after ANOVA for multiple comparisons.

**Figure 2:**
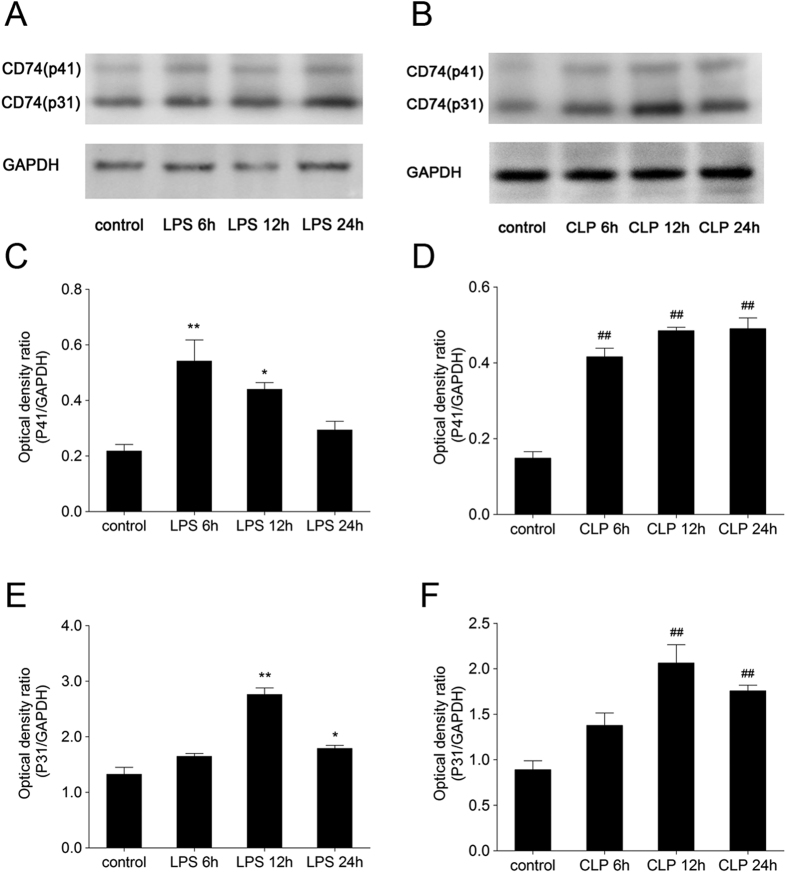
Western blot and densitometry analysis of CD74 protein in lungs. Immunoblotting using 30 μg lung proteins revealed marked up-regulation of CD74 at certain time post injury in lungs from lipopolysaccharide and cecal ligation and puncture induced acute lung injury models compared to control. Two bands of 31 and 41 kDa corresponding to two different isoforms of CD74 (p31 and p41) are shown (**A,B**). The amount of p31 expression was much higher than p41. Relative protein levels were quantified by densitometry and expressed as optical density ratio (**C–F**) with GAPDH serving as internal standards. Immunoblotting data are representative of experiments performed in triplicate and statistical differences are noted (*p < 0.05, **, ^##^p < 0.01 compared to control with Dunnett-*t* test after ANOVA for multiple comparisons).

**Figure 3:**
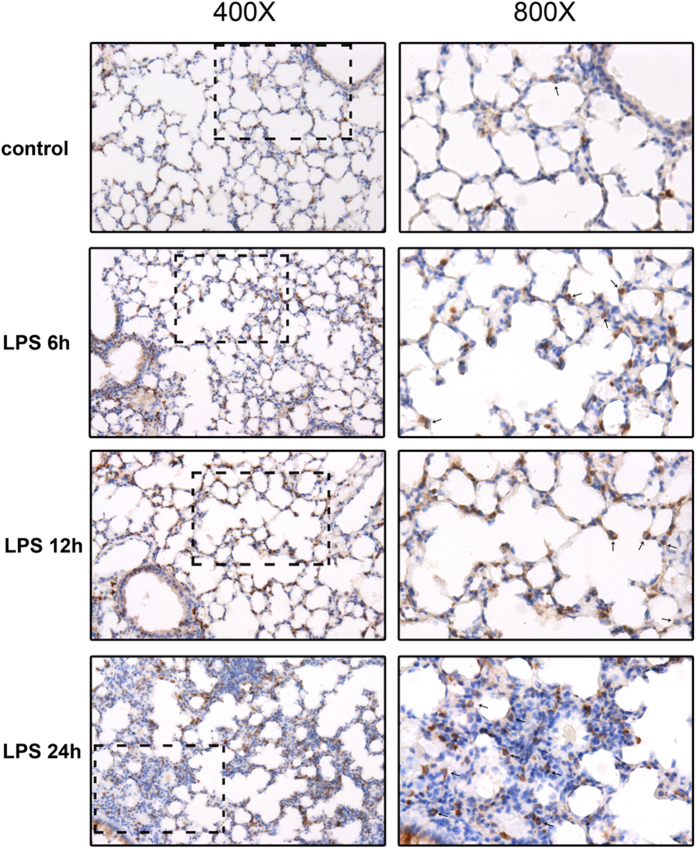
Immunohistochemistry of CD74 staining in lungs. Immunohistochemical examination was performed for CD74 in control mouse lung tissue and lipopolysaccharide induced acute lung injury model (6 h, 12 h, 24 h post lipopolysaccharide instillation). CD74 is indicated by brown staining and nuclei are counterstained in blue. Limited CD74 staining was observed in control mouse lung tissue. Increased CD74 expression in lung tissue of acute lung injury was observed. Some CD74 staining was localized on cell membrane of nucleated cells (arrow). Left magnification ×400, Right magnification ×800.

**Figure 4:**
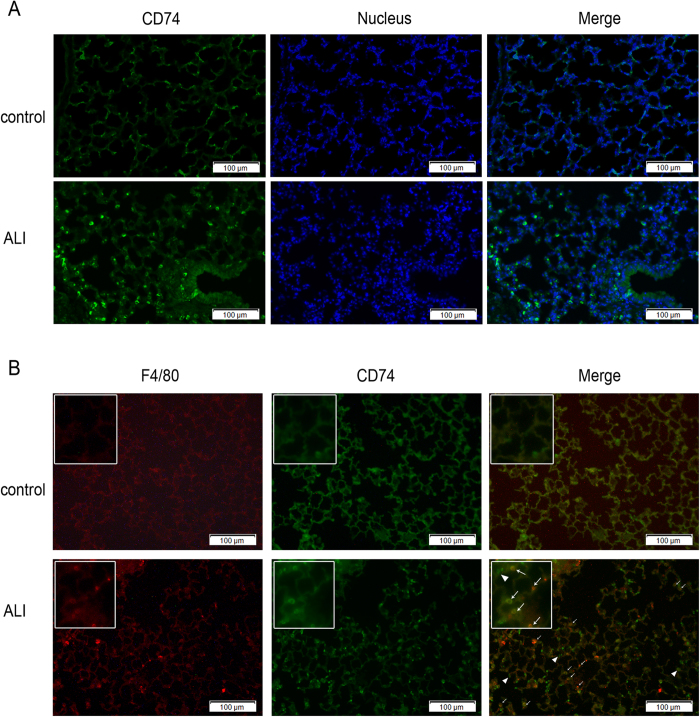
Immunofluorescence staining of CD74 in lungs. Immunofluorescence examination was performed for CD74 in control mouse lung tissue and lipopolysaccharide induced acute lung injury model. Increased surface CD74 expression (green) in lung tissue of acute lung injury was observed compared with control (**A,B**). Surface CD74-positive cells were observed mainly on the alveolar septa, and colocalize with F4/80-positive cells (macrophage cells; red) (**B**). Arrows, positive staining of macrophage cells; arrowhead, positive type II alveolar epithelial cells. Scale bar represents 100 μm.

To determine whether CD74 content is increased in the mouse lung following direct and indirect injury, the CD74 expression was examined over time. Compared to basal pulmonary CD74 expression, quantitative RT-PCR assay revealed that lungs in mice with ALI showed increased CD74 expression at 12, 24 hr after instillation of LPS (Fig. 1A). Confirmatory experiments utilizing semi-quantitative immunoblotting were aimed at correlating protein and mRNA expression levels (Fig. 2). There are two different CD74 isoforms in mice (p31 and p41) and p31 is the predominant form^18^. Compared to control mice, lungs from mice with ALI due to LPS instillation markedly increased CD74 protein expression (Fig. 2A) and densitometry of the p31 bands suggested a significant increase at 12, 24 hr post instillation, peeking at 12 hr (Fig. 2E). And the p41 bands also showed a significant increase at 6, 12 hr post instillation, peeking at 6 hr (Fig. 2C). Similarly, immunohistochemistry assay showed that CD74 expression was augmented in injured lung tissues (Fig. 3), and immunofluorescence surface staining of cells confirmed increased surface levels of CD74 after LPS instillation (Fig. 4A). Moreover, we also confirmed that CD74 staining was localized on cell surface of macrophages after double staining mouse lung tissues with anti-CD74 antibody and anti-F4/80 antibody (a macrophage marker) (Fig. 4B, also see Supplementary Fig. S1, arrows). And the cells only stained with anti-CD74 antibody were more likely alveolar type II epithelial cells (Fig. 4B, also see Supplementary Fig. S1, arrowhead). Similar results were found in CLP induced ALI mouse model that the CD74 expression in lungs increased post CLP compared to controls. (Figs 1B and 2B,D,F)

### Serum and BALF concentrations of sCD74 were increased in murine models of ALI

**Figure 5:**
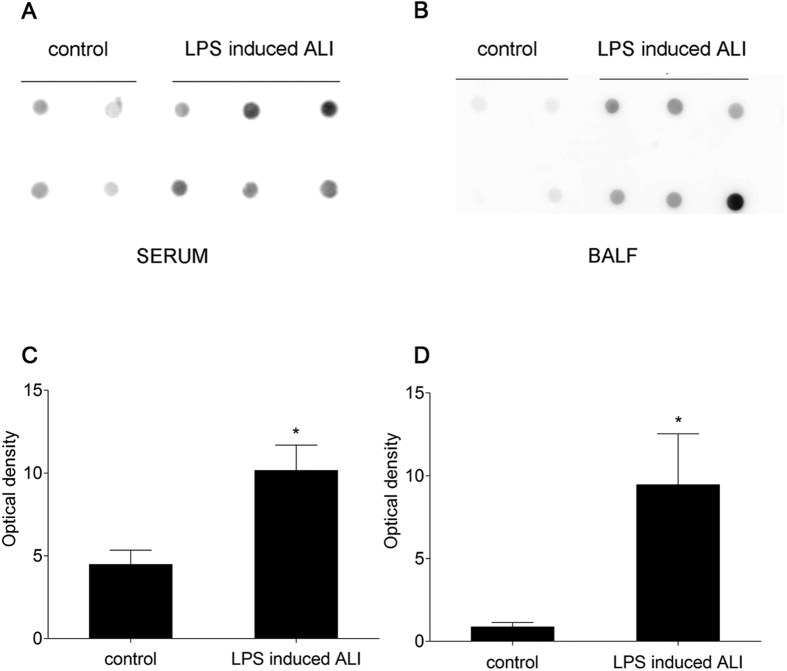
Representative dot blots for sCD74 in serum and bronchoalveolar lavage fluid from randomly selected mice. sCD74 was strongly positive in both serum and bronchoalveolar lavage fluid in the mice with acute lung injury. In contrast, sCD74 was negative in some of the control serum samples and was absent in control bronchoalveolar lavage fluid samples. (**A**: 10 serum samples; **B**: 10 bronchoalveolar lavage fluid samples) Relative protein levels were quantified by densitometry and expressed as optical density ratio (**C,D**). Data are presented as mean ± SEM, *p < 0.05 compared to control with Student *t* test.

**Figure 6:**
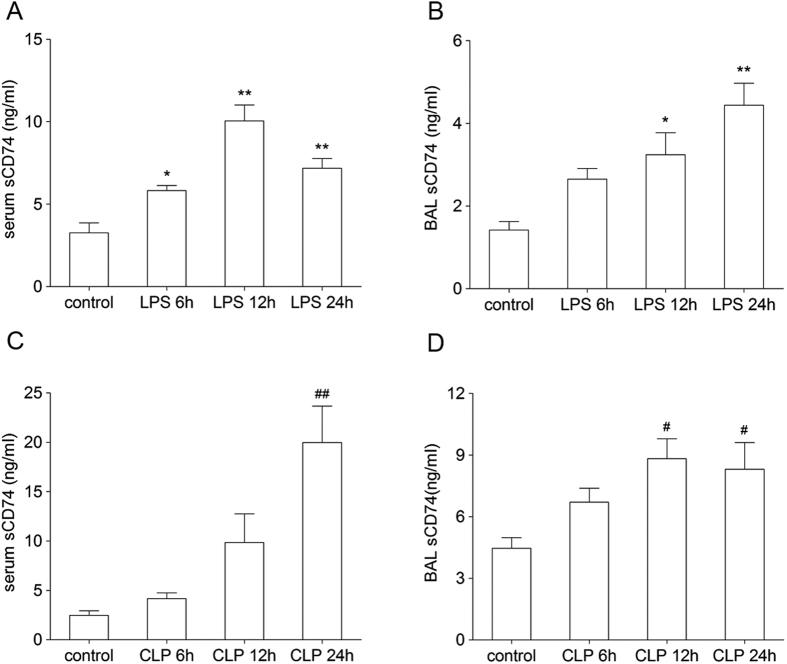
Enzyme-linked immunosorbent assay (ELISA) analysis of sCD74 concentrations in serum and BALF. sCD74 levels were measured by ELISA in the serum (**A,C**) and BALF (**B,D**) from LPS and CLP induced ALI at 6, 12, 24 hours post injury. n = 5 in each group. Data are presented as mean ± SEM, *, ^#^p < 0.05 and **, ^##^p < 0.01 compared to control with Dunnett-*t* test after ANOVA for multiple comparisons.

To test the presence of sCD74 in mice under ALI condition, we first determined sCD74 by immunoblotting using serum and BALF samples. Ten randomly selected serum and BALF samples from the *in vivo* experimental groups were examined to identify sCD74 protein by Dot blotting (Fig. 5). In mice with ALI, sCD74 was strongly positive in serum and BALF in all samples. In contrast, in control mice, sCD74 was absent in some of serum samples and all BALF samples, indicating the presence of sCD74 in both circulation and alveolar spaces in ALI. To further examine the induction of sCD74, we developed a two antibody, competitive sandwich ELISA to detect sCD74 in serum and BALF from the murine models of ALI. We observed a significant increase in the mean level of sCD74 in mice with ALI when compared to controls (Fig. 6). sCD74 in serum significantly increased at 6, 12, 24 hr after LPS instillation and peaked at 12 hr (Fig. 6A, control: 3.27 ± 0.27 ng/ml; 6 h: 5.83 ± 0.13 ng/ml; 12 h 10.06 ± 0.43 ng/ml; 24 h: 7.18 ± 0.26 ng/ml), which has a similar trend to CD74 expression. In addition, BALF was also assessed for sCD74. sCD74 in BALF significantly increased at 12, 24 hr and peaked at 24 hr (Fig. 6B, control: 1.42 ± 0.09 ng/ml; 6 h: 2.65 ± 0.11 ng/ml; 12 h: 3.24 ± 0.24 ng/ml; 24 h: 4.44 ± 0.24 ng/ml).

In CLP induced ALI model, sCD74 in serum was significantly increased at 24 hr post CLP compared with controls (Fig. 6C, 19.97 ± 3.7 ng/ml vs. 2.47 ± 0.47 ng/ml). The level of sCD74 in BALF was 4.47 ± 0.51 ng/ml in the control group and significantly increased at 12, 24 hr post CLP (12 h: 8.82 ± 0.97 ng/ml; 24 h: 8.31 ± 1.3 ng/ml) (Fig. 6D).

**Figure 7:**
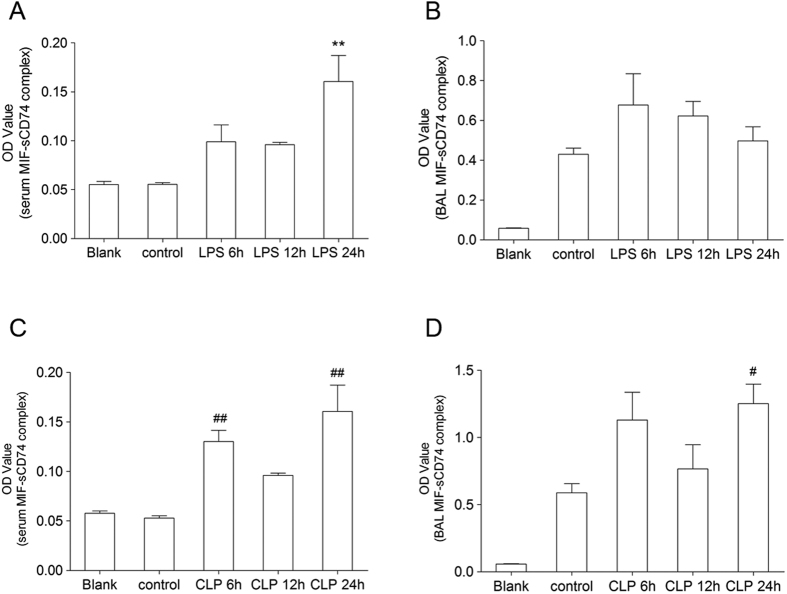
ELISA analysis of MIF-sCD74 complexes levels in serum and BALF. MIF-sCD74 levels were measured by ELISA in the serum (**A,C**) and BALF (**B,D**) from LPS and CLP induced ALI at 6, 12, 24 hours post injury and quantified by optical density. n = 5 in each group. Data are presented as mean ± SEM, *, ^#^p < 0.05 and **, ^##^p < 0.01 compared to control with Dunnett-*t* test after ANOVA for multiple comparisons.

In addition, we also detected the presence of MIF-sCD74 complexes in serum and BALF by ELISA assay. Due to lack of recombinant mouse MIF-sCD74 protein, we compared the optical density (OD) values between injured groups and control group. In both LPS and CLP induced ALI models, OD values of MIF-sCD74 in serum was significantly increased at 24 hr post injury (Fig. 7A,C). OD values of MIF-sCD74 in BALF from CLP induced ALI model was significantly increased at 24 hr (Fig. 7D). Taken together, our results suggested that circulating CD74 may exist in both monomeric sCD74 and complexic MIF-sCD74 form.

### BALF sCD74 positively correlated with lung permeability and inflammation in murine models of ALI

**Figure 8:**
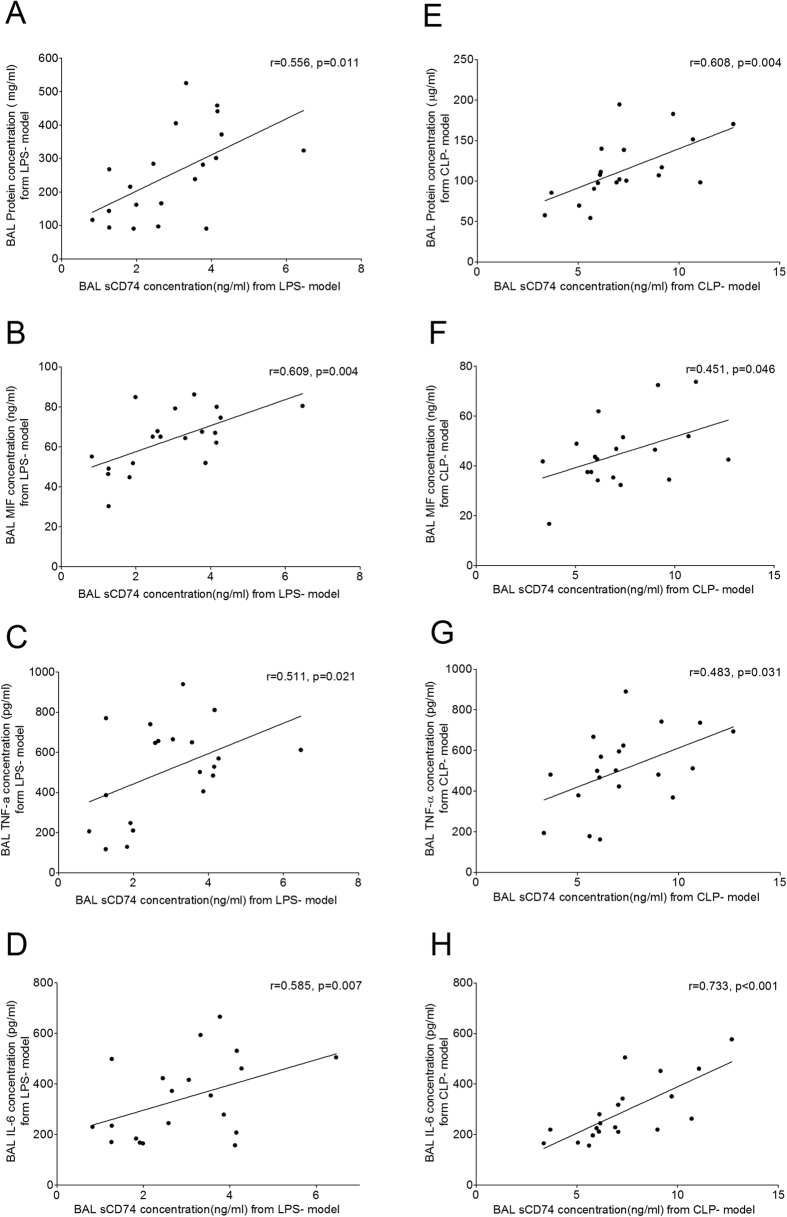
Correlations between sCD74 and total protein concentration, MIF, TNF-α and IL-6 in BALF. sCD74, total protein concentration, MIF, TNF-α and IL-6 in BALF from lipopolysaccharide and cecal ligation and puncture induced ALI were measured by ELISA assay. The increase in BALF sCD74 levels were compared to total protein concentration (**A,E**), MIF (**B,F**), TNF-α (**C,G**) and IL-6 (**D,H**) levels; n = 20 in all groups. Pearson correlation coefficients were used to analyze the relationship between sCD74 levels, total protein concentration, MIF, TNF-α and IL-6 levels in BALF.

To determine whether sCD74 is associated with severity of ALI, we compared sCD74 levels with BAL protein concentrations (a measurement of lung permeability), MIF and another two inflammatory cytokines, which are indicators of severity of lung injury. In LPS induced lung injury, a close correlation was observed between BAL protein concentrations and sCD74 levels (r = 0.556, p < 0.05, Fig. 8A). BALF MIF levels, a potential biomarker of ALI, also positively correlated with sCD74 levels (r = 0.609, p < 0.05, Fig. 8B). In addition, TNF-α and IL-6 levels, indicators of lung inflammation, significantly correlated with sCD74 release (r = 0.511 and 0.585, p < 0.05, Fig. 8C,D), suggesting that increased sCD74 levels could partly reflect inflammation of lung injury. As shown in Fig. 8E~H, the CLP induced ALI showed similar results.

### sCD74 was secreted by macrophages under MIF stimulation

**Figure 9:**
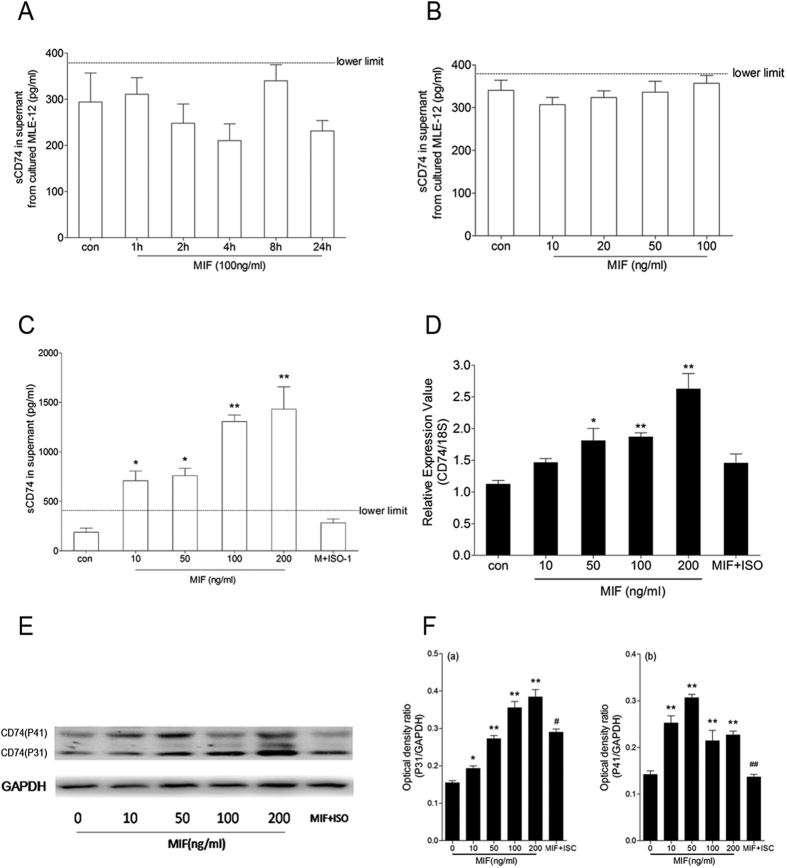
Identification of sCD74 in supernatants of culture cells following stimulation with MIF. (**A**) MLE-12 cells were treated with 100 ng/ml MIF or control media for different times (1, 2, 4, 8, 24 hrs). Then supernatants were collected and sCD74 concentrations were measured by ELISA. (**B**) MLE-12 cells were treated with different concentrations of MIF (10, 50, 100, 200 ng/ml) or control media for 24 hrs. (**C**) RAW264.7 cells were treated with different concentrations of MIF (10, 50, 100, 200 ng/ml) or control media for 24 hrs. In experiment of ISO-1 treatment, RAW264.7 cells were pre-treated with 100 μg/ml ISO-1 for 30 min, following stimulated with 100 ng/ml MIF for 24 hrs. After stimulation, supernatants were collected and subjected to ELISA assay for sCD74. After 24 hrs supernatants were collected and sCD74 concentrations were measured by ELISA. mRNA (**D**) and proteins (**E**) were collected and subjected to quantitative real-time PCR and Western blot assays, respectively. Relative protein levels were quantified by densitometry and expressed as optical density ratio with GAPDH serving as internal standards (**F**). Bar graphs represent the mean ± SEM of three independent experiments. *P < 0.05 and **P < 0.01 compared to control with Dunnett-*t* test after ANOVA for multiple comparisons.

Our above experiments and previous work showed that CD74 was expressed in both cytoplasm and cytoplasmic membrane of pulmonary macrophages and type II alveolar epithelial cells (AEC-II). To determine the cell source of sCD74, we stimulated RAW264.7 and MLE-12, used as models of pulmonary macrophages and type II alveolar epithelial cells, with different concentrations of LPS and rmMIF for certain time. As shown in Fig. 9A,B, the sCD74 level was below the detection limit of ELISA in the supernatant of MLE-12 cells under LPS and rmMIF stimulations as well as the medium of RAW264.7 under LPS stimulation, whereas sCD74 was detected in the supernatant of RAW264.7 cells under rmMIF stimulation. Moreover, a dose-dependent secretion of sCD74 response to rm-MIF was observed in our experiment (Fig. 9C). These results indicated that macrophage was one of cell sources of sCD74.

**Figure 10:**
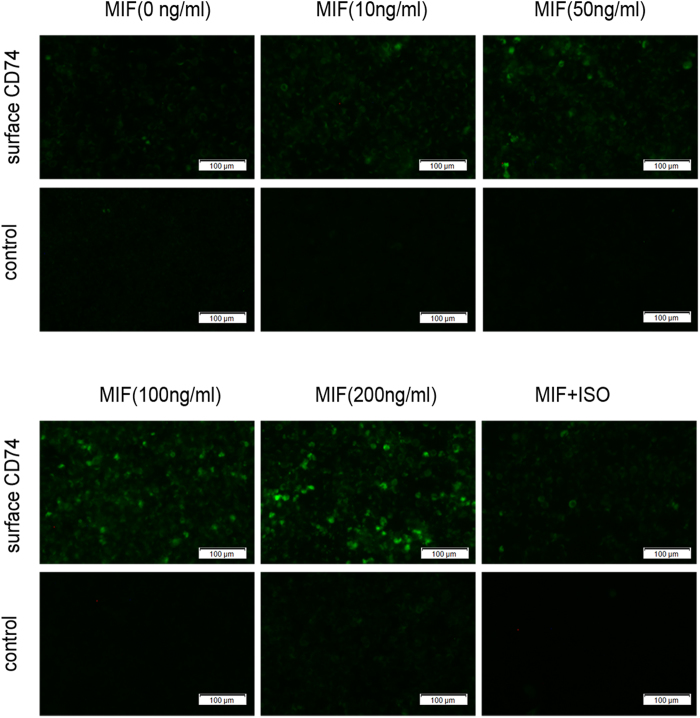
Immunofluorescence staining of surface CD74 in RAW264.7 cells. RAW264.7 cells were treated with different concentrations of MIF (0, 10, 50, 100, 200 ng/ml) for 24 hrs. In experiment of ISO-1 treatment, RAW264.7 cells were pre-treated with 100 μg/ml ISO-1 for 30 min, following stimulated with 100 ng/ml MIF for 24 hrs. After stimulation, immunofluorescence examination was performed for CD74. Surface CD74 expression was observed to increase in a concentration-dependent manner.

To elucidate whether elevation of sCD74 correlated with increasing in intracellular and surface expression of CD74, we examined the expression of CD74 gene and protein in RAW264.7 cells under rmMIF stimulation. As shown in Fig. 9D, quantitative RT-PCR assay revealed that MIF significantly increased the mRNA expression of CD74 in RAW264.7 cells in a concentration-dependent manner, with a maximum increase at a concentration of 200 ng/ml. Further western blot assay showed that MIF stimulation also resulted in significant increase in total CD74 protein expression (Fig. 9E,F). Similarly, immunofluorescence surface staining of RAW264.7 cells revealed increased surface levels of CD74 after MIF stimulation (Fig. 10, also see Supplementary Fig. S2). These results suggested that an increase in CD74 expression correlated with increased surface CD74, and in turn correlated with sCD74.

**Figure 11:**
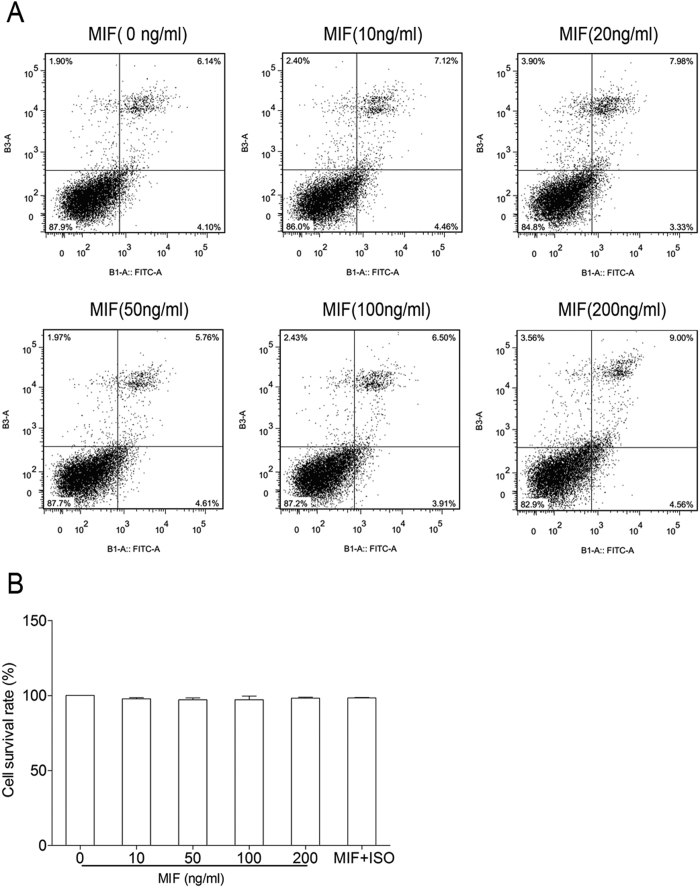
Effect of MIF on cell viability and apoptosis of RAW264.7 cells. RAW264.7 cells were treated with different concentrations of MIF (0, 10, 20, 50, 100, 200 ng/ml) for 24 hrs. In experiment of ISO-1 treatment, RAW264.7 cells were pre-treated with 100 μg/ml ISO-1 for 30 min, following stimulated with 100 ng/ml MIF for 24 hrs. Flow cytometry assay was used to examine the apoptosis of cells and Cell Counting Kit-8 was used to measure cell viability. Bar graphs represent the mean ± SEM of three independent experiments.

To further test whether the increase in sCD74 is a consequence of cell death or apoptosis, the cell viability and apoptotic response of RAW264.7 cells relative to MIF stimulation were examined. As shown in Fig. 11A, the ability of MIF to induce apoptosis was relatively low, with apoptotic indexes of 5~9% at different concentrations as measured by flow cytometry after 24 h of exposure, which was not significant compared to ~6% in the controls. The effects on cell viability are also presented, and the data showed that cell survival rate was not reduced by different concentrations of MIF compared with that in controls (Fig. 11B). These results suggested that the production of sCD74 was not due to cell death or apoptosis of macrophages.

### Recombinant mouse sCD74 stimulated increasing TNF-α and MIP-2 releases from macrophages

**Figure 12:**
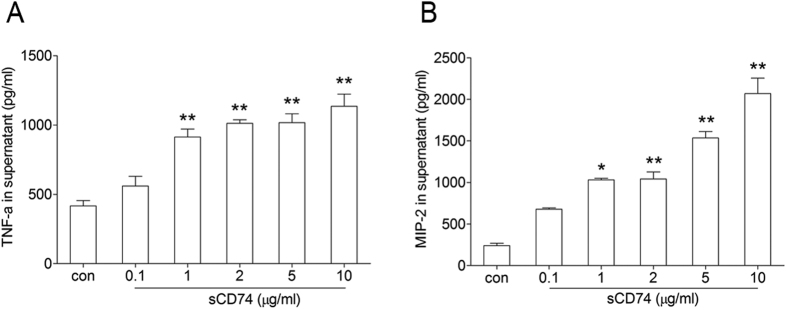
Effect of sCD74 on RAW264.7 cells. RAW264.7 cells were treated with different concentrations of sCD74 (0.1, 1, 2, 5, 10 μg/ml) or control media for 24 hrs. After 24 hrs supernatants were collected and TNF-α (**A**) and MIP-2 (**B**) concentrations were measured by ELISA. Bar graphs represent the mean ± SEM of three independent experiments. *P < 0.05 and **P < 0.01 compared to control with Dunnett-*t* test after ANOVA for multiple comparisons.

**Figure 13:**
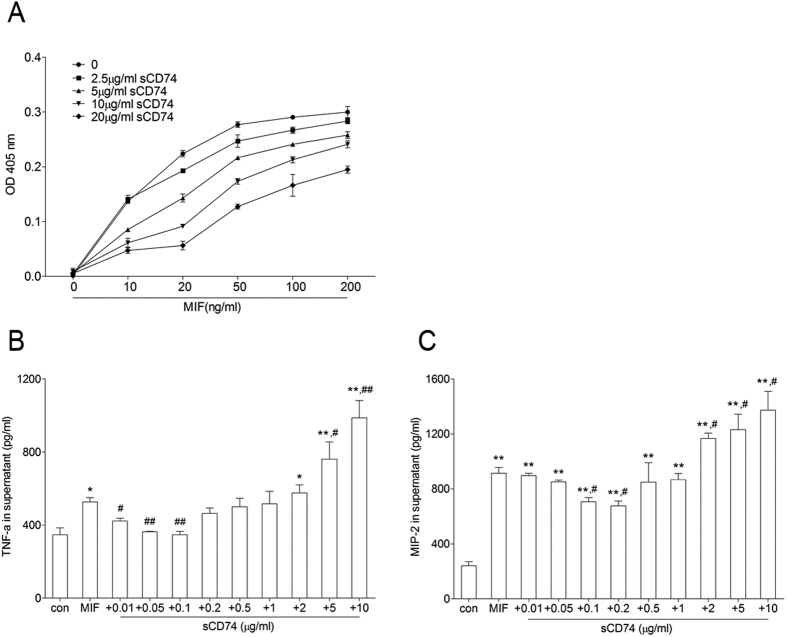
Effect of sCD74 on MIF bioactivity. (**A**) Recombinant mouse sCD74 inhibits MIF detection by ELISA method. (**B,C**) RAW264.7 cells were treated with control media and 100 ng/ml MIF with or without the presence of different concentrations of rmsCD74 for 24 hrs. After 24 hrs supernatants were collected and TNF-α and MIP-2 concentrations were measured by ELISA. Bar graphs represent the mean ± SEM of three independent experiments. *P < 0.05 and **P < 0.01 compared to control with Dunnett-*t* test after ANOVA for multiple comparisons, and ^#^P < 0.05 and ^##^P < 0.01 compared to MIF group with same statistical methods.

After we confirmed the presence of sCD74 and found its relationship with lung inflammation, we suspected that sCD74 is not only a pathologic characteristic of ALI, but that it may also play a critical role in inflammation. To investigate the bioactivity of sCD74, a recombinant mouse CD74^56–215^-Fc protein (sCD74 analogue, rmsCD74) was synthesized as previous report^6^ in our study. Firstly, we measured TNF-α and MIP-2 in culture medium of RAW264.7 under rmsCD74 stimulation (100 ng/ml). When the concentration of rmsCD74 was increased from 0.1 μg/ml to 10 μg/ml, TNF-α and MIP-2 levels were also increased in a dose-dependent manner (Fig. 12). 10 μg/ml of rmsCD74 increased TNF-α and MIP-2 levels to about two-fold and eight-fold higher than control value, respectively. However, when the concentration of rmsCD74 was under 0.1 μg/ml, neither TNF-α nor MIP-2 was detected in the supernatants by ELISA assay (data not shown). Secondly, we tested whether rmsCD74 could neutralize MIF activity as Assis *et al*. report^16^. To verify a binding interaction between rmsCD74 and MIF, we tested its ability to inhibit MIF recognition by an ELISA system. As shown in Fig. 13A, similar to Leng *et al*.^6^, the addition of rmsCD74 inhibited MIF detection in a dose-dependent fashion. We next assessed the ability of rmsCD74 to neutralize MIF activity by measuring MIF stimulated release of TNF-α and MIP-2 in RAW264.7 cell culture supernatants. The concentrations of MIF-sCD74 complexes in supernatants were also measured **(see** Supplementary Fig. S3). As shown in Fig. 13B,C, when the concentration of rmsCD74 was at a low level (10–~200 ng/ml), MIF stimulation induced release of TNF-α and MIP-2 decreased in a dose-dependent manner, suggesting sCD74 has the ability of neutralizing MIF-induced inflammation through binging to MIF. However, when the addition of rmsCD74 exceeded ~2μg/ml, TNF-α and MIP-2 concentrations in the culture media significantly increased compared to control and MIF groups. We interpreted this to be that when large doses of sCD74 were present, the remaining sCD74 played its own role in promoting inflammation in addition to those binding with the MIF.

### Serum sCD74 concentrations are increased in human ARDS and are associated with worse clinical outcomes

Serum sCD74 protein concentrations were investigated in 81 ARDS subjects and 58 healthy volunteers with the baseline demographics and clinical variables listed in Table 1. Of the 81 ARDS patients, the average age was 48.02 ± 16.05 and 54.3% were male. Inhalation injury was the major primary etiology of lung injury (46 cases), followed by trauma (35 cases). Median length of ICU stay (LOS) was 23 days (IQR, 15–44). 14 of the 81 patients died (17.3%) during their hospital stay. Comparisons of demographic and clinical severity data between surviving and non-surviving patients with ARDS are also shown in Table 1. The two groups were demographically similar in age and gender. When compared with survivors, non-survivors were more likely to suffer inhalation injury and receive mechanical ventilation, and had higher AHACHE II score, lower FiO_2_/PO_2_ ratio and fewer unassisted ventilation days.

**Figure 14:**
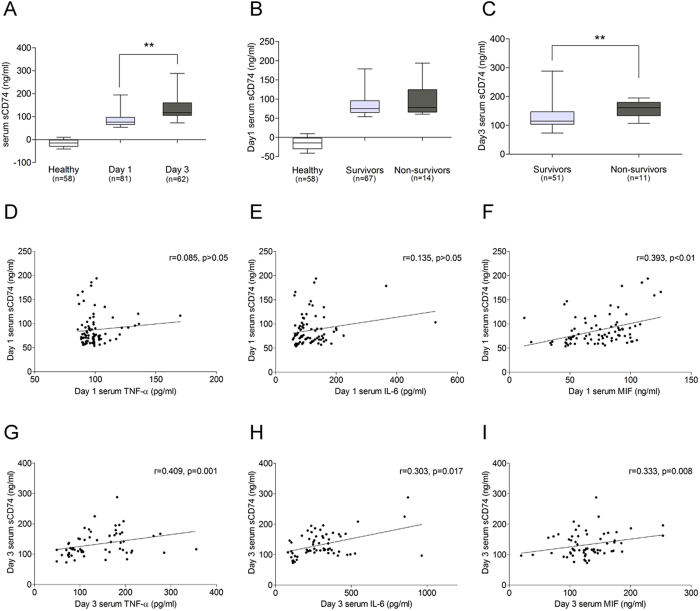
Serum sCD74 levels in ARDS patients. Serum sCD74 levels were measured in patients with ARDS (81 patients with Day 1 serum samples and 62 patients with Day 3 serum samples) as well as healthy volunteers (**A**). Data were presented as median (IQR), **p < 0.01 compared to control. Mann-Whitney U test was used to analyze difference of serum sCD74 levels between Day 1 and Day 3, and survivors and nonsurvivors. The increase in Day 1 and Day 3 serum sCD74 levels were compared to TNF-α (**D,G**), IL-6 (**E,H**) and MIF (**F,I**). Spearman rank correlation was used to analyze the relationship between sCD74 and TNF-α, IL-6 and MIF levels.

In patients with ARDS, serum sCD74 levels were 75.83(65.76, 96.36) ng/ml at day 1 and 117.0(105.2, 160.1) ng/ml at day 3, whereas in the healthy volunteers, most of serum sCD74 levels remained below the detection limit (Fig. 14A). Unexpectedly, although survivors and non-survivors had similar initial inflammatory parameters, including MIF, TNF-α, IL-6 and Day 1 serum sCD74 (Table 1 and Fig. 14B), the Day 3 serum sCD74 levels in non-survivors were significantly higher than those in survivors (160.9(133.7, 179.3) vs 115(103.9, 147.0) ng/ml, respectively; p < 0.01) (Fig. 14C). Furthermore, Day 1 serum sCD74 levels did not correlate with TNF-α and IL-6, but correlated with MIF (Fig. 14D–F). A significant correlation was observed between sCD74 levels and all the other three inflammatory cytokines (Fig. 14G–I).

We used multivariate linear regression analyses to assess the predictive value of serum sCD74 levels for ventilator-free days (Table 2). When controlling for multiple demographic and clinical variables, higher Day 3 serum sCD74 levels remained independently predictive of worse outcome. On average, there were 0.11 more days on ventilator for each 1 ng/ml increase in Day 3 serum sCD74.

**Figure 15:**
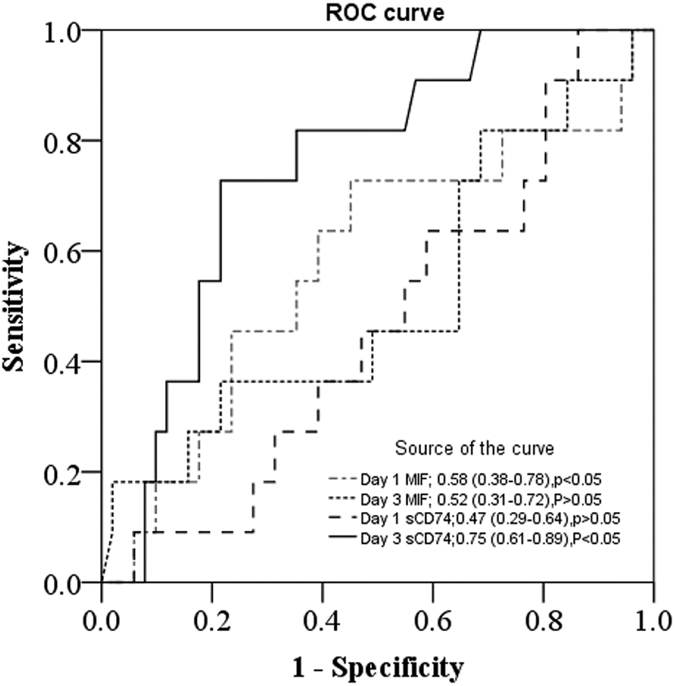
Receiver operating characteristic (ROC) curve analysis of serum MIF or sCD74 levels to predict mortality. ROCs were constructed to assess the sensitivity and specificity of serum MIF or sCD74 levels in serum from ARDS patients in predicting mortality. Areas under the curve (AUC) were calculated. Data are presented as ACU (95% Confidence interval).

ROC curves and AUC (Fig. 15) showed that Day 3 serum sCD74 levels were a better predictor of mortality than MIF and Day 1 serum sCD74 levels. The significance for Day 3 serum sCD74 was p < 0.05 (AUC: 0.75; 95% CI: 0.61–0.89), for Day 1 serum MIF was p < 0.05 (AUC: 0.58; 95% CI: 0.38–0.78), for Day 3 serum MIF was p > 0.05 (AUC: 0.52; 95% CI: 0.31–0.72) and for Day 1 serum sCD74 was P > 0.05 (AUC: 0.47; 95% CI: 0.29–0.64). And we used the Youden index (J) to select the cutoff point of serum sCD74 level for the prediction of mortality (Day1 = 118 ng/ml and Day3 = 151 ng/ml).

**Figure 16:**
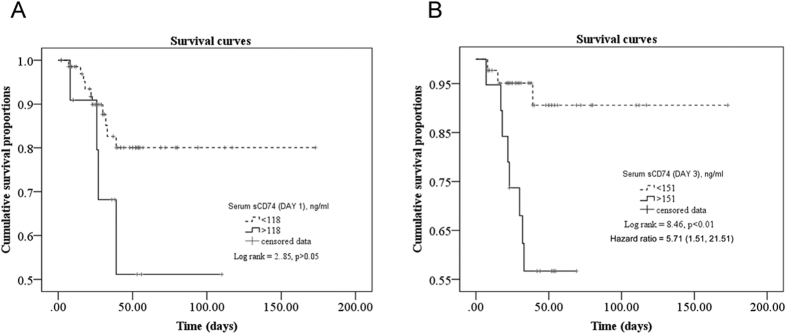
Survival curves using serum sCD74 levels higher or lower than cutoff. The survival rate of ARDS patients with high Day 1 sCD74 levels in serum (>118 ng/ml) or Day 3 serum sCD74 levels (>151 ng/ml) was compared with that of patients with low Day 1 and Day3 serum sCD74 levels, respectively. Kaplan-Meier curves and log-rank test calculations were performed to display the impact of serum sCD74 levels on survival.

Survival analysis showed that patients with Day 3 serum sCD74 >151 ng/ml had higher mortality than patients with lower levels (hazard ratio = 5.71; 95% CI, 1.51 to 21.51; P < 0.01) (Fig. 16B). However, survival analysis revealed no significant difference between patients with Day 1 serum sCD74 <118 ng/ml and those with higher levels (p > 0.05) (Fig. 16A).

Furthermore, the association between higher levels of serum sCD74 and mortality was assessed in a multivariate model (Tables 3 and 4). In the model, we included age, gender, mechanical ventilation, FiO_2_/PO_2_, Apache II score and serum sCD74 levels. Finally, only Day 3 serum sCD74 levels >151 ng/ml were associated with a higher mortality risk when we controlled for gender, FiO_2_/PO_2_ ratio and APACHE II score (OR = 6.72; 95% CI, 1.435–31.4; p = 0.016).

## Discussion

There is abundant evidence supporting that surface interaction of MIF with CD74 leads to activation of several signaling transduction regulating inflammation and immunity^6^,^18^,^19^. Recently, the soluble form of CD74 was characterized in patients with autoimmune liver disease^16^. However, no data have been provided regarding the involvement of sCD74 in ALI/ARDS. In present study, our results indicate that [1] CD74 expression was augmented in lung tissues in direct and indirect ALI murine models; [2] ALI in mice induced by LPS and CLP resulted in an increase of sCD74 levels in the serum and BALF; [3] in the ALI murine models, increased BALF sCD74 levels positively correlated with the lung permeability and inflammation; [4] MIF could stimulate sCD74 release from macrophages *in vitro*; [5] sCD74 itself has the ability of stimulating production of inflammatory cytokines; [6] ARDS patients showed significantly higher serum sCD74 levels than normal persons; [7] Serum sCD74 levels were significantly associated with worse clinical outcomes. Overall, these findings suggest the existing of sCD74 in ALI/ARDS and its potential role in reflecting severity of ALI/ARDS.

The expression of CD74 has been found to be up-regulated in several inflammatory diseases, autoimmune diseases and cancers^20^,^21^,^22^,^23^. Because of limitations in investigating the difference of CD74 in patients with ARDS, we firstly examined the expression of CD74 in lung tissues of ALI murine models induced by LPS and CLP. By real-time PCR and western blot assays, we found increased expression of CD74 in lung tissues. There are two CD74 isoforms in mice (p31 and p41) and four in humans (p43, p41, p35, p33)^24^. The short form is the predominant one in both species as previous observation estimating (in a B-lymphocyte cell line) the ratio of 9:1^25^. Our results also showed the expression of short form p31 was higher than the long form p41. In addition, we also observed intense CD74 staining in murine lungs with LPS induced lung injury by immunohistochemistry and immunofluorescence.

In addition to its intracellular form as the Class II invariant chain, CD74 has been shown to express on the cell surface independently of class II in diverse cell types^12^. Beswick *et al*.^26^ detected the surface expression of CD74 on gastric epithelial cell lines by flow cytometry and found CD74 surface expression was increased by approximately 40% in N87 and HS-738 cells and 60% in Kato III cells as a result of the IFN-stimulation. In Meyer Siegler *et al*.’s^27^ research, CD74 was found to localize to the bladder urothelial cell surface and that cell-surface expression is increased with Substances P treatment. In our experiments, immunohistochemical and immunofluorescence assays showed positively stained for CD74 on cell surface of lung macrophages, which is similar with Takahashi *et al*.^14^. Their flow cytometric analysis revealed that 24% of lung macrophages surface exhibited CD74 expression. In addition, Marsh *et al*.^15^ described CD74 is highly expressed on the cell surface of AEC-II but not AEC-I, which can be a new marker helpful to discriminate AEC-II from AEC-I. In a recent work, Sauler *et al*.^13^ found that CD74 was absent at baseline in pulmonary endothelial cells but could be induced by hyperoxia. Notably, we observed an intriguing finding of increased surface CD74 expression in pulmonary macrophages in ALI lungs. Combined with previous report that surface CD74 participates in MIF-induced pulmonary inflammatory response^14^, we hypothesized that a proportion of surface CD74 participate in amplifying inflammation in a positive feedback way. And the role of surface CD74 in diverse cells in ALI remains less certain and deserves further study.

In present study, we detected the presence of sCD74 in mouse serum and BALF with a novel two antibody, competitive sandwich ELISA, confirmed by immunobloting. And MIF-sCD74 complexes were also detected in serum and BALF of murine ALI models with ELISA assay. Furthermore, we showed for the first time that LPS or CLP induced lung injury led to increased sCD74 content in serum and BALF. As the concentrations of MIF, BAL total protein and inflammatory cytokines are indicators of lung injury, the positive correlation between sCD74 and BAL total protein, MIF or inflammatory cytokines suggest that the levels of sCD74 could reflect the inflammation of ALI.

In our work, sCD74 was detectable in both serum and alveolar space. Although our data showed that sCD74 levels in serum were much higher than levels in the BAL, the findings must be interpreted with caution because unclear dilution factors could affect real concentration of sCD74 in airspace^28^. It is also noteworthy that a leak of sCD74 from the alveolar space into the blood stream after destruction of the alveolar capillary barrier at the time of ALI may contribute to the rise in circulating CD74. Thus, whether sCD74 comes from extrapulmonary tissues or lung tissues needs further study. Notably, the ELISA detection of culture medium demonstrated that sCD74 was detectable in supernatant of RAW264.7 cells under MIF stimulation. In addition, our data showed that an increase in total CD74 expression correlated with increased surface CD74, and in turn correlated with sCD74 release. However, in the culture medium of MLE-12 cells, sCD74 was not detectable regardless of MIF stimulation. These results suggest that macrophage was an important cell source of sCD74.

Previous reports demonstrated that CD74 is a member of the regulated intramembrane proteolysis (RIP)-processed protein family^29^. In the endocytic compartments, CD74 is processed by a series of enzymes to generate a fragment of ~82 aa, which is a requirement for the second proteolytic event. The initial shedding prepares the substrate for the intramembrane proteolysis, such that the transmembrane region becomes accessible to the intramembrane endopeptidase signal peptide peptidase–like 2A (SPPL2A)^30^, which then releases CD74 intracellular domain from the lipid bilayer towards the cytosol, and that a portion of this proteolytic product translocates to the nucleus to elicit biological responses. Assis *et al*.^16^ reported that the human sCD74 in serum from autoimmune liver disease might be released after processing by proteolysis from hepatic stellate cell. In present study, our data indicate that MIF could not reduce cell viability or induce remarkable apoptosis, as opposed to higher sCD74 levels released due to cell death. On the contrary, it is possible that the production of mouse sCD74 in ALI was probably in part due to an active shedding of carboxyl terminus of cell surface CD74 locating on plasma membrane of pulmonary macrophages. However, the precise mechanism responsible for creating the sCD74 peptide remains to be elucidated.

After confirming the presence of sCD74 under ALI condition, we suspected that it may also play a critical role in inflammation. We assessed the function of sCD74 and found that sCD74 could alone stimulate the production of inflammatory cytokines from macrophage. Macrophages are important inflammatory cells correlated to the initiation of inflammatory reaction by producing multiple pro-inflammatory cytokines involved in the pathogenesis of ALI^31^. Assis *et al*.^16^ previously reported circulating CD74 could inhibit MIF-dependent ERK1/2 phosphorylation in human primary skin fibroblasts, suggesting it may neutralize MIF pro-inflammatory activity. However, we also found increasing levels of TNF-α and MIP-2 in supernatant of RAW264.7 cells after sCD74 stimulation. A possible explanation is that sCD74 has two-way regulation on inflammation, a hypothesis warranting further studies of the effect of sCD74 in the pathogenesis of inflammatory disease. In addition, the *in vivo* experiment of using murine models may better reveal the functional characterization of sCD74.

Previous studies have identified significant MIF mRNA and protein levels within the lungs of ARDS patients^2^,^5^. In addition, we also found sCD74 levels were increased in patients with ARDS and positively correlated with other inflammatory cytokines, which is consistent with our animal experiments. And from our clinical study, we found that elevation in serum sCD74 was independently associated with fewer ventilator-free days. Multiple logistic regression analysis showed that serum sCD74 levels were associated with mortality even after we controlled for potentially confounding factors. No clear correlation between concentrations of sCD74 and length of ICU stay were found in this study. Notably, soluble CD74 is also detected in serum in other conditions, thus we think it cannot be used as an ideal biomarker for ARDS but as a potential predictor of prognosis of ARDS. As a small number of patients were enrolled in this study, further multicenter clinical investigations are warranted to confirm our findings.

Despite the promising findings shown in our study, there are some limitations. We examined functional characterization of sCD74 *in vitro* using macrophages cell line RAW264.7, and it’ll be better to use primary pulmonary macrophage cells and whole animal models. More work is needed to understand the mechanism by which sCD74 is released into the air spaces and circulation under ALI condition and clarify the form of sCD74. Although the ectodomain shedding of CD74(Ii) in the endocytic compartments processed by RIP and SPPL2A was recently found as the key intramembrane protease^30^,^32^,^33^, further studies are needed to determine whether sCD74 is the extracellular segment of cell surface CD74 as a result of RIP process. Furthermore, the relationship of sCD74 release to lung inflammation, particularly in relationship to MIF, deserves further study. And additional studies of larger groups of patients with BALF samples are needed to better determine the role of sCD74 in ARDS.

In summary, the results of this study indicate that, in experimentally induced lung injury, pulmonary CD74 expression and sCD74 levels in serum or BALF are increased under ALI conditions, and sCD74 can positively modulate the release of inflammatory mediators. We also found sCD74 levels were increased in patients with ARDS, and Day 3 serum sCD74 levels >151 ng/ml was associated with severity and mortality. Taken together, all these findings suggest that sCD74 levels may be of great pathophysiological significance in ALI/ARDS. Furthermore, in the animal and human samples, quantification of sCD74 can be accomplished by ELISA. The measurement of sCD74 may stimulate more research into the pathophysiology of ALI/ARDS.

## Methods

### Regents

Cell culture reagents were purchased from Invitrogen (Carlsbad, CA, USA). The anti-CD74 antibody (Ii) for western blotting, dot blotting and immunohistochemistry was purchased from BD Pharmingen (San Diego, CA, USA), and anti-CD74 antibodies (catalog number: AF7478 and MAB7478) for enzyme-linked immunosorbent assay (ELISA) and immunofluorescence were purchased from R&D Systems (Minneapolis, MN, USA). Recombinant mouse MIF protein (rmMIF), anti-MIF antibody and ELISA kits for MIF, TNF-α, IL-6 and macrophage inflammatory protein 2 (MIP-2) were obtained from R&D Systems (Minneapolis, MN, USA). The anti-F4/80 antibody and mouse monoclonal antibody GAPDH was purchased from Cell Signaling Technology Inc (Beverly, MA, USA). Horseradish peroxidase-conjugated secondary antibodies were provided by Santa Cruz Biotechnology (Dallas, Texas, USA), and FITC-conjugated secondary antibody was purchased from Abbkine Inc (Redlands, CA, USA). Recombinant mouse sCD74 protein (CD74^56–215^-Fc, rmsCD74) was obtained from Chimerigen Inc. (Allston, MA). The MIF inhibitor (S,R)-3-(4- hydroxyphenyl)-4,5-dihydro-5-isoxazole acetic acid methyl ester (ISO-1) was purchased from EMD Chemicals Inc (San Diego, CA, USA).

### Animal studies

#### Animal models

C57BL/6 mice were purchased from Experimental Animal Center, Second Military Medical University (Shanghai, China). The animal experiments included 80 male C57BL/6 mice (8–10 weeks). In each model, forty mice were randomly divided into 4 groups (n = 10/group): control, 6 hr, 12 hr, and 24 hr injured groups. All animal experiments were approved by the Institutional Animal Care and Use Committee of the SMMU and processed according to the Guide for Care and Use of Laboratory Animals published by the US NIH (publication no. 96-01). And all the methods were carried out in accordance with approved guidelines and regulations.

LPS-induced Lung Injury Model. Mice were anesthetized with sodium pentobarbital (60 mg/kg) and 2 mg/kg LPS (*Escherichia coli* O111:B4; Sigma, Poole, UK) diluted in 50 μl of physiological saline were instilled intratracheally via a 20-gauge catheter (LPS group). Mice instilled with 50 μl physiological saline were used as the control animals (control group).

CLP-induced Lung Injury Model. In the animal model of sepsis-induced lung injury, sepsis was induced by cecal ligation and puncture (CLP) as previous report^34^. Briefly, mice were anesthetized before a 1-cm midline incision was made on the anterior abdomen, and the cecum was carefully isolated. Then, cecum was ligated just below the ileocecal valve and punctured twice with a 25-gauge needle. The abdominal cavity was then closed in two layers, followed by fluid resuscitation (CLP group). Mice proceeding same procedure without ligated and punctured were used as the control animals (CLP control group).

Groups of mice (n = 5/group) were killed at 6, 12 and 24 hr post injury before collecting blood by cardiac puncture. The blood samples were left to clot for 30 min before centrifugation for 10 min at 1000 g, and the serum was stored at −80 °C for further examination. Postmortem BALF were performed by instilling and withdrawing sterile physiological saline (0.8 ml) through a tracheal cannula using a 20 gauge Surflo i.v. catheter (Terumo, Elkton, MD). This procedure was repeated three times, and the three BALF samples were pooled and centrifuged (800 × g, 10 min), and the supernatant portions were stored at −80 °C for further examination.

Another 5 mice in each group were used to collect lung tissues to prepare slices without collecting BALF. The right lung was fixed in 10% formalin for at least 48 h and then embedded in paraffin for later histopathologic examination, and the remaining lung tissues were isolated and used to extract protein and RNA for further experiment.

### Cell culture

The murine macrophage cell line RAW264.7 and alveolar type II epithelial cells (MLE-12) were purchased from American Type Culture Collection (Manassas, VA). The RAW264.7 cells were cultured in Dulbecco’s Modified Eagles Medium (DMEM) with 10% FBS at 37 °C under a humidified atmosphere of 5% CO_2_. MLE-12 cells were cultured in HITES medium supplemented with 2% fetal bovine serum at same incubation condition. The cells were exposed to rmMIF at increasing concentrations (0, 10, 50, 100 or 200 ng/ml) for different times. Then supernatants were collected and subjected to ELISA assay. In order to determine the bioactivity of sCD74, we performed similar experiments with RAW264.7 cells cultured for exposure to recombinant mouse sCD74 (0, 0.1, 1, 2, 5 or 10 μg/ml) for 24 hrs.

### Quantitative Real Time–Polymerase Chain Reaction (qRT-PCR)

Total RNA was extracted from lungs using TRIzol reagent and reverse transcribed (RT; Qiagen). qRT-PCR was performed using StepOnePlus Real-Time PCR Systems (Applied Biosystems Inc., Foster City, CA). ΔCT analysis was used to calculate expression in comparison to18S RNA. Primers for CD74 are as follows^15^: FP- CAT GGA TGA CCA ACG CGA C; RP-TGT ACAGAG CTC CAC GGC TG.

### Western Blot Analysis

Lung tissues or cells were lysed in lysis buffer. Lysates were separated from debris by centrifugation (4 °C, 12000 r/min) for 10 min and the supernatants were collected. Protein concentration was determined by bicinchoninic acid (BCA) protein assay kit (Thermo Scientific, Rockford, IL). Then the supernatants were boiled in 5X loading buffer (Beyotime Biotechnology, China) for 5 minutes. Samples were separated on 10% of polyacrylamide gels and transferred to polyvinylidene fluoride membranes. After blotting, the membranes were blocked in Tris-buffered saline with 0.05% Tween 20 (TBST) containing 5% nonfat dry milk for 90 min at room temperature, and then, incubated with TBST containing the specific primary antibodies overnight at 4 °C. The membranes were washed with TBST, and then incubated with HRP conjugated secondary antibodies in TBST. After washing, signals were revealed using the Pierce ECL Plus Western Blotting Substrate (Thermo Scientific, Rockford, IL). Western blots were scanned and densitometry ratios normalized to GAPDH content were analyzed using ImageJ software (NIH, US).

### Dot Blotting

Mouse serum and BALF samples were diluted 1:10 in TBS, and 2 μl of each sample was spotted onto the nitrocellulose membrane by using a narrow-mouth pipet tip. Dot blots were blocked in TBST containing 5% nonfat dried milk for 1 hr. Then, the membrane was incubated with TBST containing the same rat anti-mouse CD74 antibody as the one used in the Western blot for 1 hr at room temperature. The membrane was washed with TBST, and then incubated with a HRP conjugated anti-rat IgG in TBST. After washing, the membrane was developed utilizing enhanced chemiluminescence reagents and scanned.

### Immunohistochemistry

The sections of lung tissues were deparaffinized and incubated with anti-mouse CD74 antibody over night at 4 °C. The sections were washed with phosphate buffered saline (pH = 7.4), and then incubated with anti-rat-IgG. Secondary labeling was achieved by using biotinylated rabbit anti-rat antibody. Horseradish peroxidase-conjugated avidin and brown-colored diaminobenzidine were used to visualize the labeling. Finally, the slides were counterstained with hematoxylin.

### Immunofluorescence

The procedures were similar to immunohistochemistry assay except for incubating with FITC-conjugated secondary antibody diluted in blocking solution for one hour at room temperature. After washing with PBS, slides were incubated with 4′,6-diamidino-2-phenylindole, dihydrochloride (DAPI) in blocking solution for 5 minutes at room temperature. Images were obtained using a LECIA DMI3000B fluorescent microscope and LECIA Application Suite software (Wetzlar, Germany).

### Total protein concentration in BALF

Immediately after collection of BALF and centrifugation, the total protein concentration in BALF was measured using Coomassie protein assay kit (Thermo Scientific, Rockford, IL).

### ELISA of TNF-α, IL-6, MIF and MIP-2

The levels of cytokines TNF-α, IL-6, MIF and MIP-2 in the BALF and supernatants were measured using mouse ELISA kits according to the manufacturer’s instructions.

### ELISA of sCD74 and MIF-sCD74

A competitive sandwich ELISA for the detection of mouse sCD74 and MIF-sCD74 was developed as part of this study. Briefly, maxisorp nunc-immuno plates (Thermo Scientific, Rockford, IL) were coated with capture antibody (sheep anti-CD74 antibody) at 4 °C overnight. Plates were washed and blocked with blocking buffer for 2 hrs, and serum, BALF, and culture medium samples from murine were added and incubated overnight. After the addition of detection antibodies (rat anti-CD74 or sheep anti-mouse MIF), the plates were incubated for 2 hrs. Horseradish peroxidase-conjugated goat anti-rat IgG or donkey anti-sheep IgG was next applied and incubated for one hour and the plate was developed with tetramethylbenzidine plus hydrogen peroxide solution. The concentration of sCD74 was determined using Elx800 (BioTek Instruments, Inc. VT), and normalization was based on concentration–response curves, using CD74 recombinant protein. The specificity and sensitivity of the ELISA assays were provided in the Supplement materials (see Supplementary Figs S4–6).

### ELISA assay of MIF binding to recombinant mouse CD74

Increasing concentrations of MIF were captured by an immobilized anti-MIF antibody, followed by the addition of the rmsCD74 and MIF detection antibody. The bound complexes were detected with Horseradish peroxidase-conjugated antibody and tetramethylbenzidine plus hydrogen peroxide solution as a substrate.

### Apoptosis measurement by flow cytometry

RAW264.7 cells were plated onto 6-well plates (10^5^cells/well) and treated with 0, 10, 20, 50, 100, 200 ng/ml MIF for 24 hrs, respectively. The apoptosis of cells was measured using Annexin V-FITC/PI apoptosis detection kit (eBioscience,San Diego, CA). Flow cytometry analysis was performed on Cytoflex (Beckman Coulter, USA).

### Cell viability measurement by Cell Counting Kit-8 (CCK-8)

*In vitro*, the RAW264.7 cells were seeded in a 96-well plate at a density of 5 × 10^3^ cells/well and treated with 0, 10, 20, 50, 100, 200 ng/ml MIF for 24 hrs, respectively. Cell viability was measured using CCK-8 (Dojindo Laboratories, Japan) according to the manufacturer’s instructions.

### Human studies

The clinical study was registered on ClinicalTrial.gov (NCT02201446) and conducted at the Changhai Hospital of the Second Military Medical University. The Ethics Committees from this institution approved the protocol of this study (No. CHEC2014-023), and informed consent was obtained from each participant or family member. All experiments were performed in accordance with relevant guidelines and regulations. Eighty-one patients were enrolled consecutively over a two-year time period (2014–2015) and identified as ARDS prospectively according to the Berlin definitions of the European Society of Intensive Care Medicine and American Thoracic Society on ARDS^35^. Exclusion criteria were an age of less than 16 years, pregnancy, chronic obstructive pulmonary disease according to medical history and failure to obtain informed consent. Fifty-eight healthy volunteers recruited from the general population who had no significant medical history and no medications were categorized as control patients. A 5-ml sample of blood was obtained on Day 1 and Day 3. Nineteen patients did not have blood collected on Day 3. Therefore, 62 patients underwent assay of Day 3 serum sCD74 levels. The samples were immediately transferred to the central laboratory and centrifuged, and the serum was extracted and stored at −80 °C.

Clinical data collection included demographics, primary etiologies of ARDS, inflammation variables and clinical variables for calculation of APACHE II (Acute Physiology and Chronic Health Evaluation II). Information for all above variables was collected within the first 24 hr of enrollment into this study. Ventilator-free days were calculated as previously described^36^.

### Statistical Analysis

Statistical analyses were done using IBM SPSS Statistic 21 software. Data were represented as mean ± SEM or median and interquartile ranges (IQR), and statistical comparisons of the results were made using Students *t* test, ANOVA, Mann-Whitney U test or χ^2^ test. Pearson correlation coefficients were used to compare markers of disease severity with BALF sCD74 levels. Receiver operating characteristic curve analysis was carried out to determine the area under the curve (AUC). Kaplan-Meier curves and log-rank test calculations were performed to display the impact of serum sCD74 levels on survival. Multiple logistic regression analyses were carried out to determine the association between serum sCD74 levels and mortality. Statistical significance was defined as p < 0.05.

## Additional Information

**How to cite this article**: Wu, G. *et al*. Relationship between elevated soluble CD74 and severity of experimental and clinical ALI/ARDS. *Sci. Rep.* **6**, 30067; doi: 10.1038/srep30067 (2016).

## Supplementary Material

Supplementary Information

## Figures and Tables

**Table 1:** Clinical characteristics of ALI patients and healthy volunteers.

	Healthy volunteers(n = 58)	**Total patients**	Non-survivors(n = 14)	Survivors(n = 67)	p-value*
Age	39.52 ± 10.43	48.02 ± 16.05	50.14 ± 20.6	47.58 ± 15.08	>0.05
Male gender	32	44	6	38	>0.05
Attributable causes of ALI					
Trauma	N	35	1	34	**<0.01**
Inhalation injury		46	13	33	
AHACHE II	N	14.35 ± 6.39	19.79 ± 6.45	13.21 ± 5.81	**<0.01**
Mechanical ventilation (Y/N)	N	51/30	14/0	37/30	**<0.01**
Days of unassisted ventilation (IQR)	N	22 (8.5, 28)	7.5 (2.5, 14.75)	26 (17, 28)	**<0.01**
FiO_2_/PO_2_	N	2.1 ± 0.46	1.73 ± 0.38	2.17 ± 0.44	**<0.01**
Day 1 MIF (ng/ml)	N	73.64 ± 23.27	83.31 ± 25.2	71.62 ± 22.53	>0.05
Day 3 MIF (ng/ml)#	N	129.27 ± 41.12	137.14 ± 52.64	127.58 ± 38.63	>0.05
Day 1 TNF-α (IQR, pg/ml)	N	97.09 (92.32, 100.82)	98.39 (93.26, 100.69)	96.6 (92.32, 103.11)	>0.05
Day 3 TNF-α (pg/ml)#	N	138.67 ± 64.26	138.834 ± 45.36	138.63 ± 68.02	>0.05
Day 1 IL-6 (IQR, pg/ml)	N	101.63 (79.5, 138.9)	124.63 (91.74, 144.68)	91.86 (75.48, 136.67)	>0.05
Day 3 IL-6 (pg/ml)#	N	305.36 ± 177.14	264.62 ± 57.02	314.154 ± 192.84	>0.05
ICU length (IQR)	N	23 (15, 44)	24.5 (16.5, 32.25)	23 (14, 48)	>0.05

^*^Non-survivors compared with Survivors.

^#^Only 62 patients with Day 3 blood samples were analyzed.

**Table 2:** Linear regression analysis of serum sCD74 and ventilator-free days in ARDS.

**Variables**	**Study day**	**Univariate**	**p Value**	**Multivariate**	**P Value**
MIF	Day 1	−0.13 (−0.22, −0.03)	0.01	−0.08 (−0.18, 0.02)	>0.05
MIF	Day 3	−0.04 (−0.11, 0.02)	>0.05	−0.01 (−0.08, 0.05)	>0.05
sCD74	Day 1	−0.01 (−0.09, 0.06)	>0.05	0.006 (−0.07. 0.08)	>0.05
sCD74	Day 3	**−0.10 (−0.17, −0.04)**	**0.001**	**−0.11 (−0.19, −0.03)**	**0.007**

Multivariate analysis included age, gender, etiology, APACHE II score, FiO_2_/PO_2_, TNF-α, and IL6.

**Table 3:** Multiple binomial logistic regression analysis of serum sCD74 (day 1) to predict mortality.

**Variables**	**Odds ratio**	**95% confidence interval**	**P value**
Age	1.007	0.96–1.05	0.766
Female gender	7.88	0.98–63.17	**0.052**
Mechanical ventilation	>999		0.997
FiO_2_/PO_2_	0.11	0.01–0.97	**0.046**
Apache II	1.24	1.03–1.49	**0.024**
Serum sCD74 >118 ng/ml	13.21	0.83–209.79	**0.067**

Eighty-one patients with enough Day 1 serum samples were analyzed.

**Table 4:** Multiple binomial logistic regression analysis of serum sCD74 (day 3) to predict mortality.

**Variables**	**Odds ratio**	**95% confidence interval**	**P value**
Age	0.98	0.93–1.04	0.481
Female gender	17.17	1.34–220.24	**0.029**
Mechanical ventilation	>999		0.998
FiO_2_/PO_2_	0.04	0.00–0.68	**0.025**
Apache II	1.23	0.99–1.51	**0.059**
Serum sCD74 >151 ng/ml	10.50	1.08–102.03	**0.043**

Sixty-two patients with enough Day 3 serum samples were analyzed.
