# Supplementary material for: Relationship between elevated soluble CD74 and severity of experimental and clinical ALI/ARDS
Source: Sci Rep. 2016 Jul 22;6:30067. doi: 10.1038/srep30067 (PMC4957083; doi:10.1038/srep30067)
Supplement: Supplementary Information [file srep30067-s1.pdf]

Supplementary Information

Relationship between elevated soluble CD74 and severity of experimental and clinical ALI /ARDS

Guosheng Wu#, Yu Sun#, Kang'an Wang#, Zhengli Chen, Xingtong Wang, Fei Chang, Ting Li, Ping Feng, Zhaofan Xia

Supplementary Figure S1

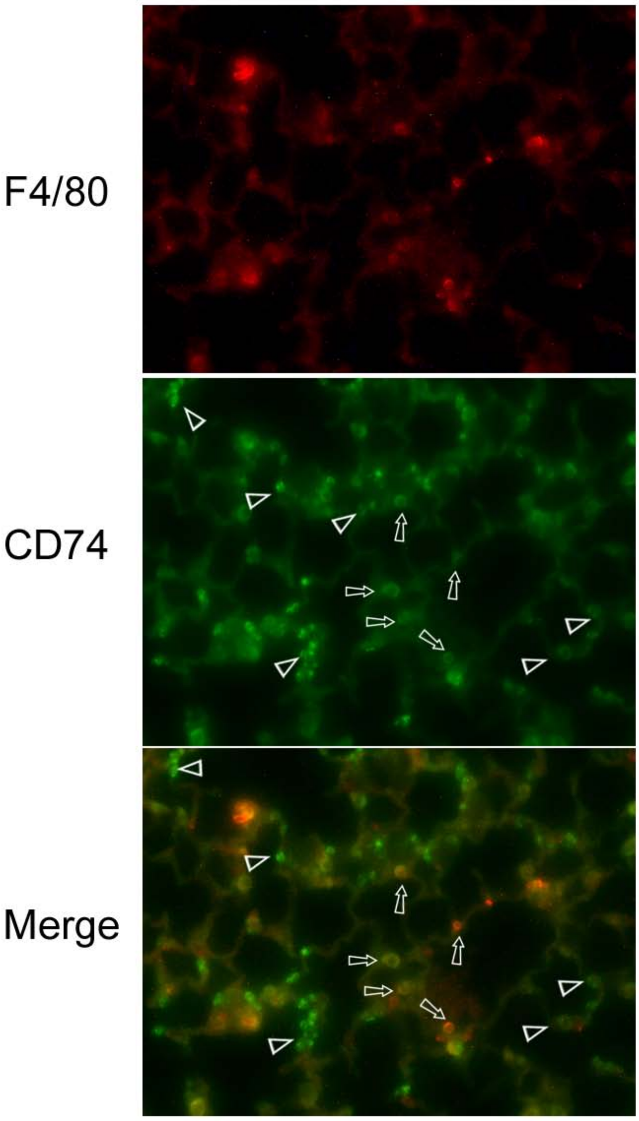

Supplementary Figure S2

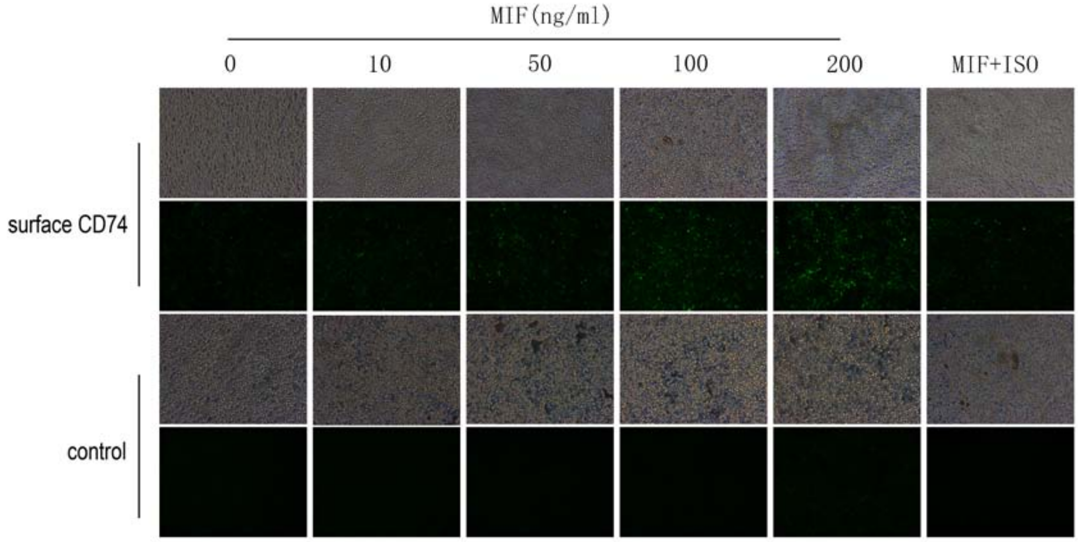

Supplementary Figure S3

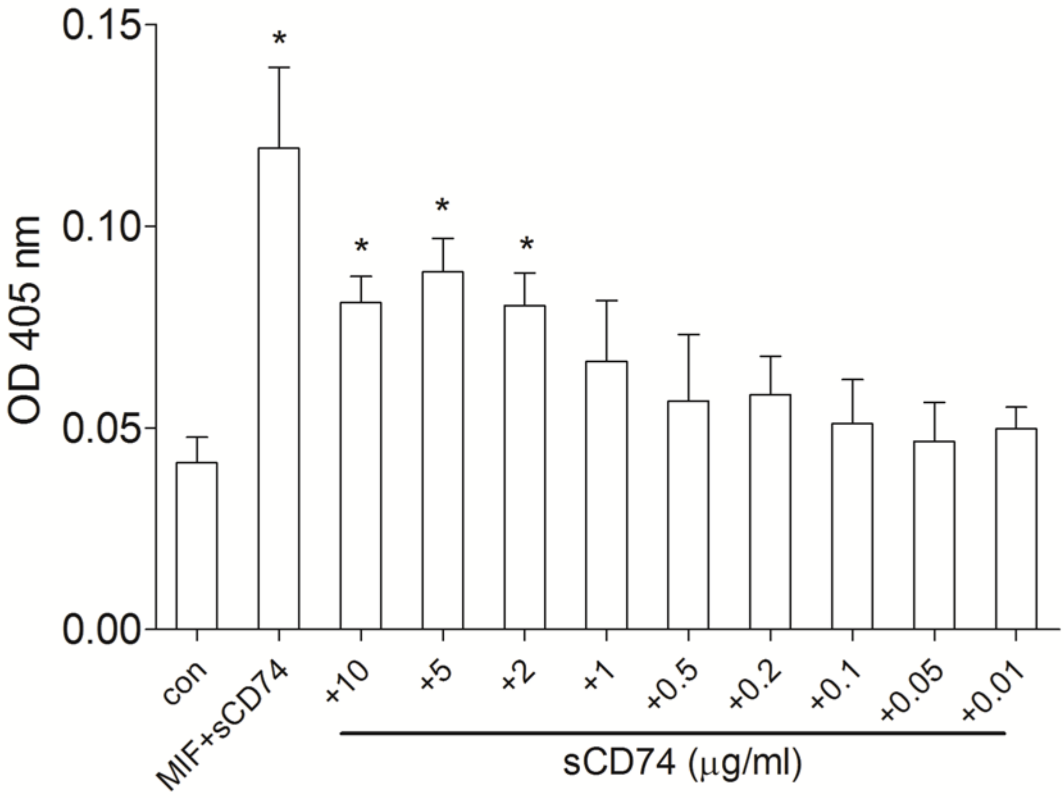

Supplementary Figure S4

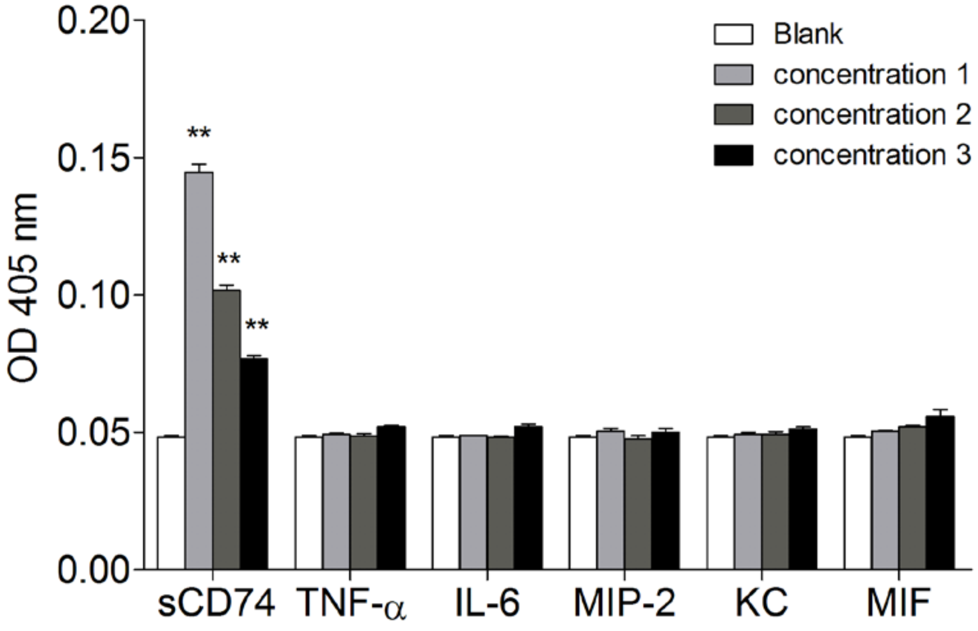

Supplementary Figure S5

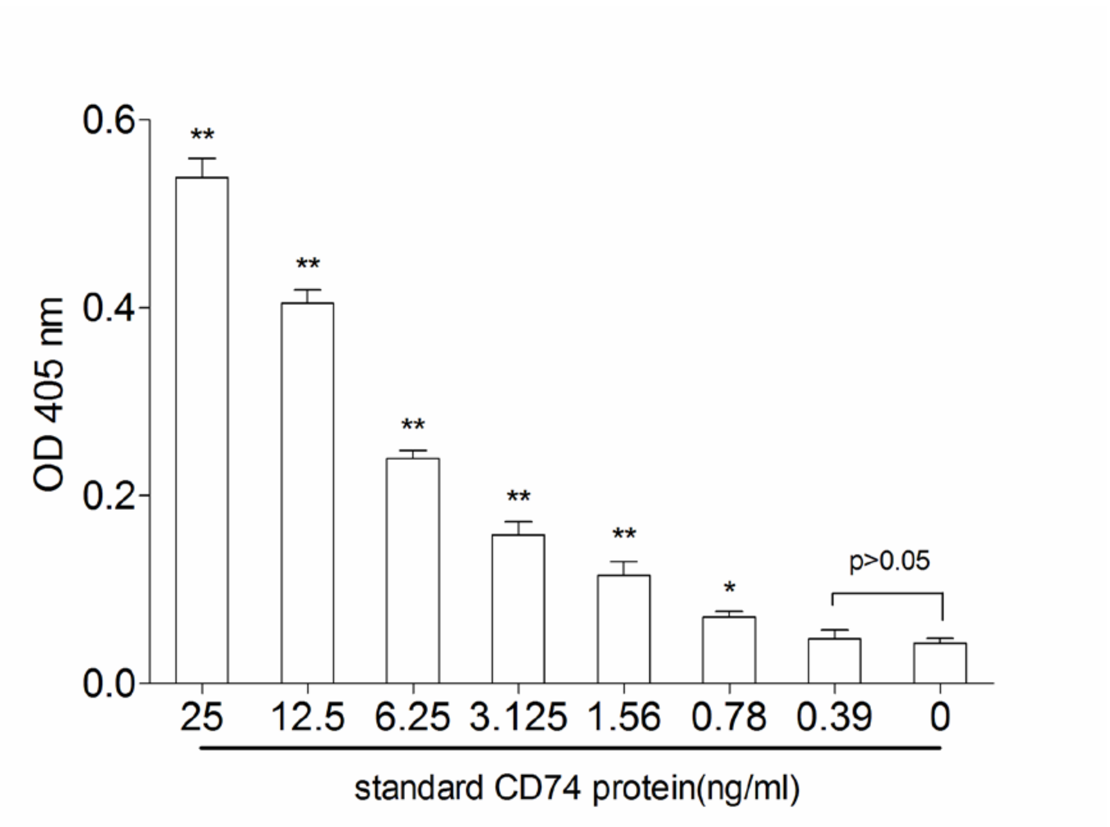

Supplementary Figure S6

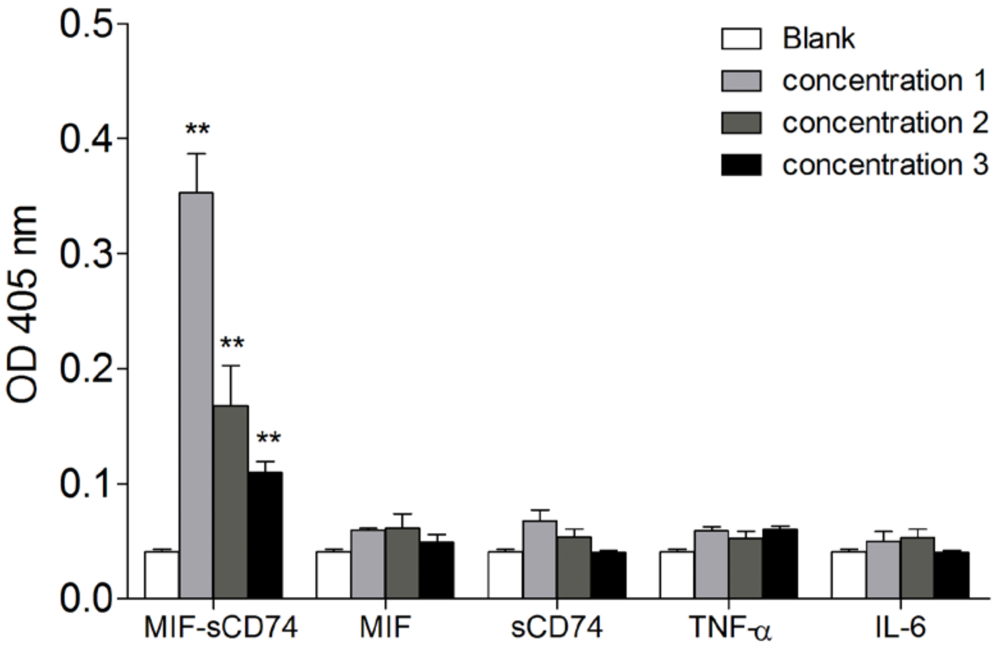

Figure legend

Figure S1. Immunofluorescence staining of surface CD74.

CD74 immunolocalization was performed by double-labeled immunofluorescence using F4/80 antibody (red) and CD74 antibody (green). Arrows, positive staining of macrophage cells; arrowhead, positive type II alveolar epithelial cells. Magnification × 400.

Figure S2. Immunofluorescence staining of surface CD74 in RAW264.7 cells.

Surface CD74 expression in RAW264.7 cells was observed to increase in a dose-dependent manner after MIF stimulation. Magnification × 200.

Figure S3. ELISA analysis of MIF-sCD74 complexes levels in supernatants.

RAW264.7 cells were treated with control media and 100ng/ml MIF with or without the presence of different concentrations of sCD74 for ~2 hrs. Supernatants were collected and MIF-sCD74 complexes were measured by ELISA. The mixture of recombinant mouse MIF protein and sCD74 protein was used as a positive control. *$P<0.05$ compared to control with Dunnett-t test after ANOVA for multiple comparisons.

Figure S4. Specificity of ELISA assay for sCD74

TNF-, IL-6, MIP-2, KC and MIF standard proteins were assayed and exhibited no cross-reactivity. Concentration 1 > Concentration 2 > Concentration 3. **$P<0.01$ compared to Blank with Dunnett-t test after ANOVA for multiple comparisons.

Figure S5. Sensitivity of ELISA assay for sCD74

2-fold dilutions of CD74 standard protein (range from 25ng/ml-390pg/ml) were made to test the detection limit. The limit is ~390pg/ml according to statistics. *$P<0.05$, **$P<0.01$ compared to 0ng/ml group with Dunnett-t test after ANOVA for multiple comparisons.

Figure S6. Specificity of ELISA assay for MIF-sCD74 complexes

MIF, sCD74, TNF-α and IL-6 standard proteins were assayed and exhibited no cross-reactivity. The mixture of recombinant mouse MIF protein and sCD74 protein was used as a positive control. Concentration 1 > Concentration 2 > Concentration 3. **$P<0.01$ compared to Blank with Dunnett-t test after ANOVA for multiple comparisons.
